# A Rare Case of Charcot-Marie-Tooth Disease Type 1C With an Unusual Presentation

**DOI:** 10.7759/cureus.8517

**Published:** 2020-06-08

**Authors:** Shaweta Khosa, Shri K Mishra

**Affiliations:** 1 Neurology, Olive View - University of California Los Angeles Medical Center, Los Angeles, USA; 2 Neurology, Keck School of Medicine of the University of Southern California, Los Angeles, USA

**Keywords:** neurology, charcot-marie-tooth, pes cavus, demyelinating diseases, sensorimotor neuropathy, genetic mutation

## Abstract

Charcot-Marie-Tooth neuropathy type 1 (CMT1) is an inherited demyelinating neuropathy characterized by distal muscle weakness and atrophy. Charcot-Marie-Tooth disease type 1C (CMT1C) is a rare form of CMT1 caused by mutations in the lipopolysaccharide-induced tumor necrosis factor (LITAF) or small integral membrane protein of the lysosome/late endosome (SIMPLE) gene. Phenotypically, CMT1C is characterized by sensory loss and slow conduction velocity, and is typically slowly progressive and often associated with pes cavus foot deformity and bilateral foot drop. A 42-year-old female presented with a 10-year history of slowly progressive bilateral calf pain and cramps. After multiple electromyography/nerve conduction studies (EMG/NCS) and genetic testing, the patient was revealed to have CMT1C with a heterozygous pathogenic variant, c.334G>A (p.Gly112Ser). However, the presentation of the patient's CMT1C phenotype was unusual compared to patients with similar diagnosis in a previous study, including a normal sensory exam with the exception of high arches and mildly reduced vibratory sense. Additionally, the patient's teenage son already started showing symptoms of CMT1C despite the fact that the onset of the disease typically occurs at an older age. This particular case further highlights the idea that the phenotype related to CMT1C may have a wide spectrum of disease severity.

## Introduction

Charcot-Marie-Tooth disease type 1C (CMT1C) is a rare form of an inherited demyelinating neuropathy, Charcot-Marie-Tooth neuropathy type 1 (CMT1). It is caused by mutations in the lipopolysaccharide-induced tumor necrosis factor (LITAF) or small integral membrane protein of the lysosome/late endosome (SIMPLE) gene [1]. This autosomal dominant disease is marked by sensory loss and slow conduction velocity, and is typically slowly progressive and often associated with pes cavus foot deformity and bilateral foot drop [2]. However, given the rarity of this condition, with a prevalence of <1/1,000,000, the exact phenotype is not very well characterized.

## Case presentation

A 42-year-old female presented with a 10-year history of slowly progressive bilateral calf pain and cramps. The patient denied paresthesias or weakness. She also denied a history of trauma in the area of the pain. She described the pain as a dull pain that limited her daily activities and reported similar complaints in her teenage son. To help with the pain, she had applied a topical cream and used compression stockings. She had also tried physical therapy but had not found it helpful.

Motor examination revealed posterior calf atrophy with normal bulk elsewhere. Examination showed normal muscle tone, strength, and reflexes in all extremities. The patient had decreased vibration sensation in her toes bilaterally with intact proprioception. Further examination revealed bilateral pes cavus with hammertoe.

A previous electromyography/nerve conduction study (EMG/NCS) performed in December of 2016 had suggested moderately-severe demyelinating sensorimotor polyneuropathy with conduction velocities ranging between 20-30 m/s. A repeat EMG/NCS was requested in 2018 as the patient’s exam did not fit the previous EMG/NCS findings. The repeat EMG/NCS showed slightly worse changes than the previous EMG/NCS study with moderate to severe demyelinating sensorimotor polyneuropathy with evidence of secondary axonal changes (Tables 1, 2). 

**Table 1:** Sensory nerve conduction study showing moderate to severe demyelinating sensorimotor neuropathy with evidence of secondary axonal changes Lat: latency; Amp: amplitude; NR: not reported

Nerve/Sites	Rec. site	Onset Lat	Peak Lat	NP Amp	PP Amp	Segments	Distance	Velocity	Temp.
		ms	ms	µV	µV		cm	m/s	°C
R Ulnar - Digit V (Antidromic)									
Wrist	Dig V	3.91	5.52	9.2	11.5	Wrist - Dig V	11	28	
						A.Elbow - Wrist			
R Radial - Anatomical Snuff Box (Forearm)									
Forearm	Wrist	2.45	3.65	23.3	16.3	Forearm - Wrist	10	41	
R Sural - Ankle (Calf)									
Calf	Ankle	NR	NR	NR	NR	Calf - Ankle	14	NR	23.3
R Superficial Peroneal - Ankle									
Lateral Leg	Ankle	NR	NR	NR	NR	Lateral Leg - Ankle	14	NR	23.4

**Table 2:** Motor nerve conduction studies showing severe demyelinating sensorimotor neuropathy APB: abductor pollicis brevis; ADM: abductor digiti minimi; EDB: extensor digitorum brevis; AH: abductor hallucis; Tib Ant: tibialis anterior; NR: not reported

Nerve/Sites	Muscle	Latency	Amplitude	Rel Amplitude	Duration	Segments	Distance	Lat Diff	Velocity	Temp.
		ms	mV	%	ms		cm	ms	m/s	°C
R Median - APB										
Wrist	APB	4.69	1.5	100	5.73	Wrist - APB	7			23.6
Elbow	APB	14.27				Elbow - Wrist	24.5	9.58	26	23.7
R Ulnar - ADM										
Wrist	ADM	5	5	100	11.56	Wrist - ADM	7			23.6
B.Elbow	ADM	11.61	2.9	58.4		B.Elbow - Wrist	19.5	6.61	29	23.6
A.Elbow	ADM	14.22	3.1	105		A.Elbow - B.Elbow	9	2.6	35	23.4
						A.Elbow - Wrist		9.22		23.4
R Peroneal - EDB										
Ankle	EDB	NR	NR	NR	NR	Ankle - EDB	8			23.3
						Pop Fossa - Ankle		NR		
R Tibial - AH										
Ankle	AH	6.3	2.6	100	7.66	Ankle - AH	8			23.4
Pop Fossa	AH	NR				Pop Fossa - Ankle				23.4
R Peroneal - Tib Ant										
Fib Head	Tib Ant	5.16	4.3	100	11.61	Fib Head - Tib Ant	12			23.4
Pop Fossa	Tib Ant	7.5	5.2	121	12.34	Pop Fossa - Fib Head	8	2.34	34	23.6

**Figure 1:**
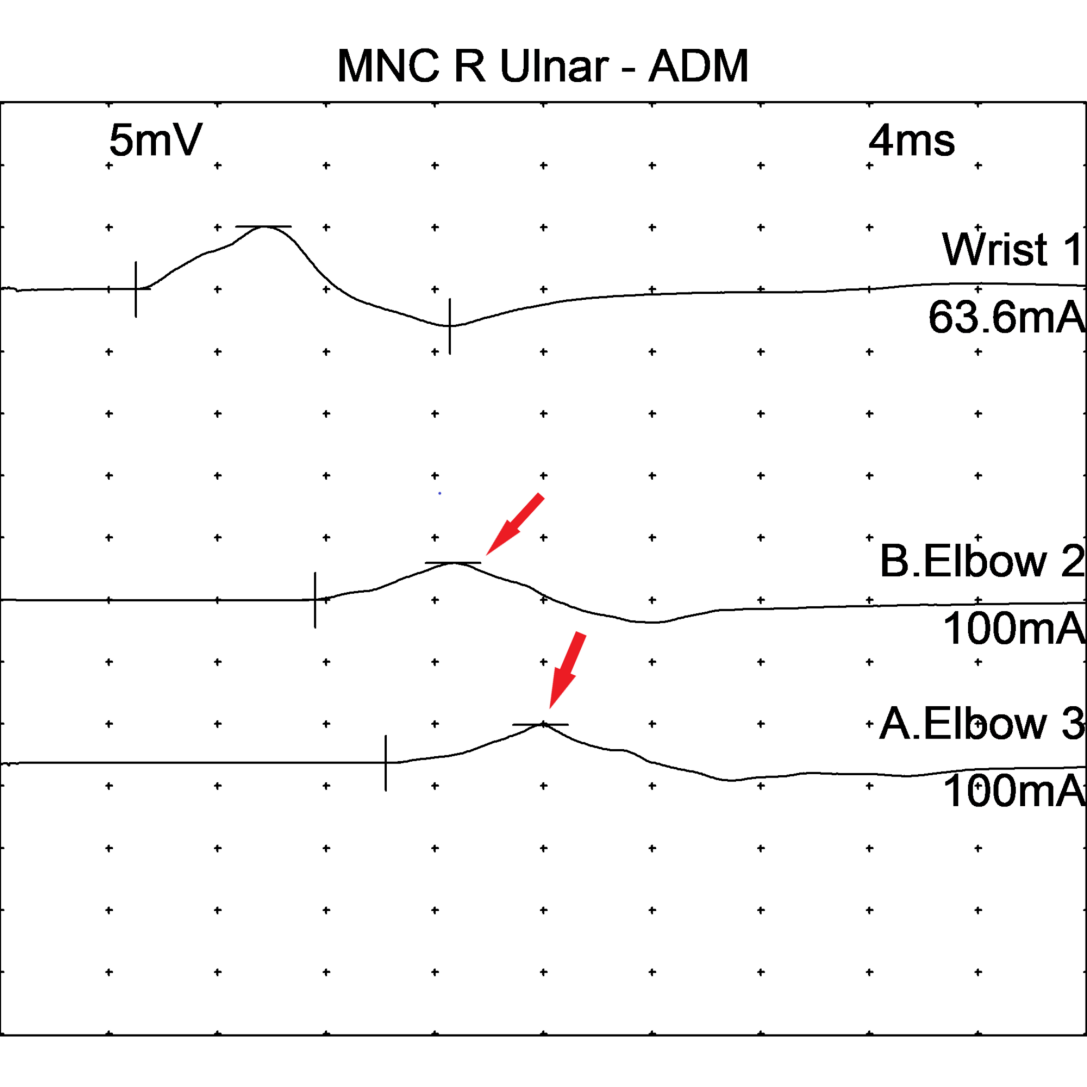
Motor nerve conduction studies in the right ulnar nerve - abductor digiti minimi showing decreased conduction velocities and a 2 mv drop in amplitude across the elbow (arrows) MNC: motor nerve conduction; ADM: abductor digiti minimi

**Figure 2:**
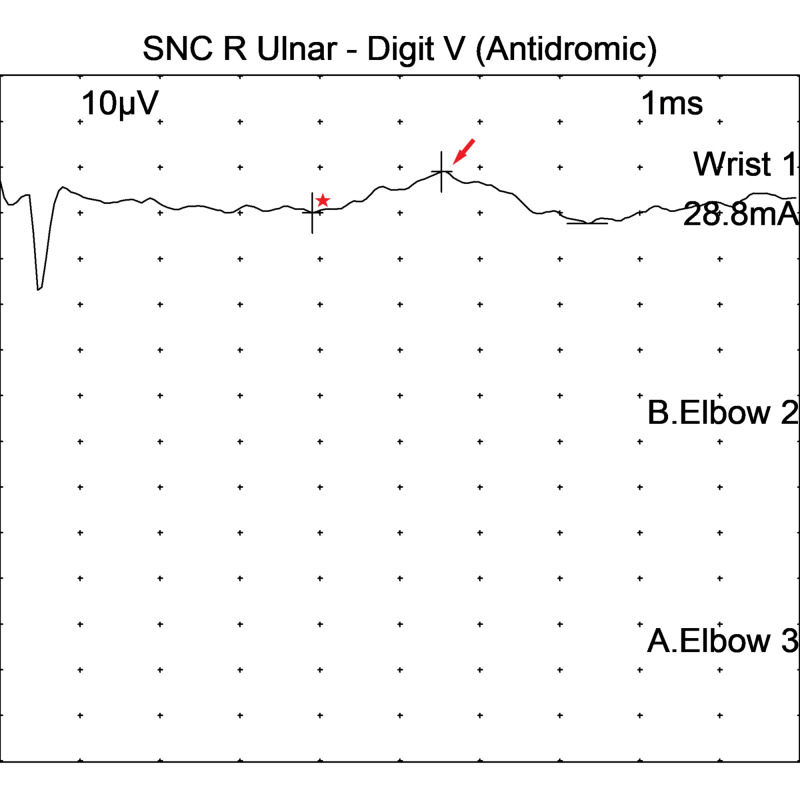
Sensory nerve conduction studies in the right ulnar sensory nerve showed prolonged peak latency (*) and decreased amplitude (arrow) SNC: sensory nerve conduction

EMG findings on motor NCS in the right ulnar nerve are shown in Figure 1, while sensory NCS from the right ulnar nerve can be seen in Figure 2. 

A repeat EMG/NCS performed one year later showed that previously present sensory responses were absent. The findings were suggestive of hereditary motor and sensory neuropathy. Furthermore, MRI and CT scans of the lower extremities were unremarkable.

Genetic testing using sequence analysis revealed a heterozygous pathogenic variant, c.334G>A (p.Gly112Ser) (rs104894519), in LITAF consistent with the diagnosis of CMT1C. Another heterozygous variant of uncertain significance, c.559C>T (p.Arg187Cys) (rs768929130, ExAC 0.2%), was also identified in the periaxin gene (PRX), which is associated with autosomal recessive Charcot-Marie-Tooth disease type 4F (CMT4F) [3]. The clinical significance of this second variant is uncertain at this time and is of uncertain significance. We further plan to arrange genetic testing for CMT1C in the patient’s children as her son has reported similar symptoms.

## Discussion

A combination of EMG/NCS and genetic testing revealed that the patient presented to the clinic had CMT1C, a rare form of CMT1 marked by mutations in LITAF or the SIMPLE gene. In particular, the patient was characterized by a heterozygous pathogenic variant, c.334G>A (p.Gly112Ser), in LITAF. The LITAF gene encodes for an endosomal transmembrane protein, which is highly expressed in the myelinating Schwann cells [4]. The mutation is reported to result in mislocalization of the LITAF protein from the late endosome or lysosome to the mitochondria. The severity could also vary based on this degree of mislocalization [5]. CMT1C has been described as a mild form of neuropathy in a previous small case series, including in one of 10 patients with Gly112Ser mutations in LITAF [6]. The previous small case series report two distinguishable groups of CMT1C patients. The first group labeled as “CMT-like” incorporated patients resembling the CMT phenotype such as motor weakness, distal motor deficit, and diminished reflexes. The second group labeled as “sensory form” encompassed patients with predominantly sensory symptoms or asymptomatic complaints of mild transient paraesthesia, transient pain in feet or fingers, and cramps and repeated ankle sprains. The patients had either motor weakness, diminished reflexes, or an abnormal sensory exam. However, our patient had a normal exam with the exception of high arches and mildly reduced vibratory sense. Although the symptoms usually begin in childhood in the majority of the patients, some variability in age has been observed [1,7]. Our patient presented with the symptoms in the third decade. However, our patient’s son is beginning to show symptoms at the age of 13 years. Since the patient’s son is showing symptoms at such an early age, we plan to see him in our clinic to characterize his phenotype and send for genetic testing.

This article was previously presented as a meeting abstract at the 144th Annual Meeting of the American Neurological Association on 4/2/2019.

## Conclusions

The phenotypic variation presented in our patient demonstrates additionalities of already known CMT1C-related phenotype and highlights the wide range of disease severity related to the phenotype of CMT1C. The case presented to our clinic also underlines the importance of early diagnosis for symptomatic treatment and the need for optimal genetic counseling.
